# A co-designed evaluation study to identify Breastfeeding Knowledge of General Practitioners’ and Practice Nurses’

**DOI:** 10.1093/heapro/daae021

**Published:** 2024-03-07

**Authors:** Denise McGuinness, Siobhan Ni Mhurchu, Kate Frazer, Nancy Bhardwaj, Paula Cornally, Marie Cantwell, Marina Cullen, Edel McNamara, Rita McDonald, Lisa Carroll, Walter Cullen, Regina Kincaid, Niamh Vickers

**Affiliations:** School of Nursing, Midwifery & Health Systems, Health Sciences Centre, University College Dublin, Belfield, Dublin 4, Ireland; Child Health Programme Development Officer, HSE Community Healthcare Organisation, Dublin North City & County, Tonlegee Health Centre, Dublin 5, D05 K2E6, Ireland; School of Nursing, Midwifery & Health Systems, Health Sciences Centre, University College Dublin, Belfield, Dublin 4, Ireland; School of Public Health, Physiotherapy and Sport Science, University College Dublin, Belfield, Dublin 4, Ireland; School of Nursing, Midwifery & Health Systems, Health Sciences Centre, University College Dublin, Belfield, Dublin 4, Ireland; HSE Community Healthcare Organisation, Dublin North City and County, HSE Unit 1,2,3, Nexus Building, Block 6A, Blanchardstown Corporate Park, Ballycoolin, Eircode D15 CF9K, Ireland; Rotunda Hospital Parnell Square East, Rotunda, Dublin 1, D01 P5W9, Ireland; Department of Health Promotion and Improvement, Health and Wellbeing Division, HSE Dublin North City and County Community Healthcare, 1st Floor, Unit 4 Nexus Building, Block 6A Blanchardstown Corporate Park, Dublin 15, D15 CF 9K, Ireland; Regional Centre for Nurse & Midwifery Education, Academic Centre, Connolly Hospital, Blanchardstown, Dublin 15, D15 X40D, Ireland; Rotunda Hospital Parnell Square East, Rotunda, Dublin 1, D01 P5W9, Ireland; School of Medicine, University College Dublin, Belfield, Dublin 4, Ireland; Patient Public Representative, Dublin, Ireland; School of Nursing, Midwifery & Health Systems, Health Sciences Centre, University College Dublin, Belfield, Dublin 4, Ireland

**Keywords:** breastfeeding, lactation, attitudes, knowledge, education, GP, general practice nurse, patient and public involvement (PPI)

## Abstract

The World Health Organization and American Academy of Paediatrics recommend exclusive breastfeeding until 6 months of age, with continued breastfeeding along with complementary solid foods for up to 2 years and beyond. Despite the well-established importance of breastfeeding, Irish rates remain the lowest in Europe. Healthcare professionals’ breastfeeding knowledge and skills have a positive impact on increasing breastfeeding rates. There is limited evidence of the knowledge, attitudes or practices of general practitioners (GPs) and general practice nurses (GPNs), which is essential to breastfeeding in Ireland. The aim of this study was to evaluate the breastfeeding knowledge, attitudes and practices of GPs and GPNs in one community healthcare organisation (CHO) in Ireland. A co-designed evaluation study was used following low-risk ethical exemption (LS-LR-22-161). A modified version of a validated breastfeeding questionnaire was developed. A Project Steering Committee was established that included patient, and public involvement stakeholders. The anonymised survey was distributed via online Qualtrics platform (November 2022–February 2023). STROBE Guidelines were utilised. The overall response rate was 25.9% (*n* = 121) and valid responses were reported in the article. The total population size was *n* = 468 (GPs *n* = 290 and GPNs *n* = 178). Our pilot study identified that 42.7% (*n* = 47/110) of respondents never attended a breastfeeding education programme, and 53.9% (*n* = 55/102) identified that their knowledge could be improved. The majority of respondents, 92.9% (*n* = 92/99) wish to complete further education in breastfeeding. The results of this pilot study in one CHO in Ireland indicate a gap in knowledge and a need for specific breastfeeding and lactation theoretical and skills training for GPs and GPNs working in primary care to support, promote and protect breastfeeding.

**Contribution to Health Promotion**

- There is limited contemporary evidence reporting the knowledge, attitudes or practices of GPs and GPNs which is essential to supporting breastfeeding both nationally and internationally.
- Reduced confidence exists for healthcare professionals to support lactation following a preterm birth and provide guidance for lactation suppression following infant loss/death.
- A breastfeeding and lactation module embedded in undergraduate and graduate education is suggested in preparation for GP and GPN practice.

## BACKGROUND

It is recognised that after many years of decline breastfeeding and human milk are becoming increasingly valued in biomedicine, public health and society at large (Tomori *et al.*, 2018), notwithstanding an expanded use of donor human milk globally (Cassidy and Dykes, 2019). Breastfeeding provides infants and children with the best nutrition and care in early life (Ip *et al*., 2009; Pérez-Escamilla et al., 2023), provides health benefits for mothers and has a tremendous effect on the health and well-being of entire communities (Victora *et al*., 2016; Shenker, 2020). Indeed, the economic impact of suboptimal breastfeeding is increasing globally (Campbell *et al*., 2019). The World Health Organization (WHO, 2023), American Academy of Paediatrics (Meek and Noble, 2022) and the Health Service Executive (HSE, 2019) in Ireland recommend exclusive breastfeeding until 6 months of age when solid foods are introduced, with continued breastfeeding for up to the age of 2 years and beyond. Despite well-established health, social and economic advantages to the newborn and the mother, breastfeeding practices remain low in many parts of Europe, including Ireland and in the USA (Quesada *et al*. 2020; Kinoshita *et al*., 2021) and have not achieved optimal prevalence globally (Pokhrel *et al*. 2015; Rollins *et al*., 2016; WHO, 2023).

There is a growing team of interdisciplinary healthcare providers supporting families who breastfeed including general practitioners (GPs), general practice nurses (GPNs), community nurses, children’s nurses. As primary healthcare providers GPs and GPNs are two professional groups who can provide the knowledge, skills and support that women and their families require to initiate and continue breastfeeding. In Ireland, the Maternity and Infant Care Scheme provides an agreed programme of care including two post-natal visits in primary care (HSE, 2023), where breastfeeding and lactation knowledge and skills are necessary for GPs and GPNs. In order to provide evidence-based lactation care, GPs and GPNs require theoretical and clinical skills and training. Whelan and Kearney (2015) reported gaps and inconsistencies in breastfeeding knowledge and attitudes of Irish healthcare professionals with inconsistencies in the advice provided to parents (Mc Gorrian *et al*. 2010; Kinoshita *et al*. 2021). A recent report from the Royal College of Physicians Ireland (Kinoshita *et al*. 2021) presents evidence from Finneran and Murphy (2004) highlighting that 9.1% of GPs in the mid-west region of Ireland had any training on breastfeeding, and confirming the social and economic divide in breastfeeding rates, especially for the minoritised ethnic traveller community and for lower socioeconomic and marginalised population groups.

When breastfeeding support is offered to women both the duration and exclusivity of breastfeeding are increased, in addition to reducing the number of women who stop breastfeeding after three to 4 months (Gavine *et al*., 2022). Internationally a lack of breastfeeding education, knowledge and training for medical and nursing students, including GP training programmes, confirms the impact, which limits breastfeeding support provided (Holtzman and Usherwood, 2018; Biggs *et al*., 2020; Blitman *et al*., 2022).

Women’s experiences of breastfeeding can empower or disempower depending on access and the available support (Van Esterik, 2018). Brown (2017) confirms the challenges for women in not reaching pre-determined breastfeeding goals. The associated feelings experienced by women include guilt, failure, weakness and sadness in the early post-natal period and could have consequences as women continue on their mothering journey. Jackson *et al*. (2021) report in their systematic review of evidence, women’s guilt when their breastfeeding intentions were unmet and with inevitable use of commercial milk formula (CMF).

Philip *et al*. (2023) report the established culture of combined feeding or combination feeding, offering breastmilk/breastfeeding and CMF, in Ireland, which is detrimental to the continuation of breastfeeding. The socio-cultural infant feeding norms result in consequences for health, wellbeing and increasing inequalities (Timori *et al*., 2018). Little is known about the knowledge, attitudes and practices (KAP) of GPs and GPNs in Ireland in the past decade. The aim of this pilot study was to establish a baseline of knowledge, attitudes and skills of GPs and GPNs in one Health Service Executive (HSE) community healthcare region in Ireland so that education and training supports could be developed for that geographical area.

**Aim:** To evaluate the breastfeeding knowledge, attitudes and practices of GPs and GPNs in one community healthcare organisation (CHO) in Ireland.

## METHOD

### Study design project management

A co-designed evaluation study design, using a survey method, was used. This was a collaboration between the HSE infant feeding committee subgroup in the designated CHO area and an academic team at the university. A Project Steering Committee (PSC) was established at the outset and invited members included all stakeholders thus ensuring patient and public involvement (PPI) in the co-designed study. Members of the PSC included practitioners; midwives, nurses and a GP; health service managers; academics; researchers; PPI members and three HCPs with the international board certified lactation consultant qualification. The project team met regularly as the survey was advanced to meet the research aims. A study gatekeeper was identified from the PSC to support communication and recruitment with GPs and GPNs in the CHO.

### Setting

One of nine regional CHOs in Ireland was chosen following initial discussions with HSE colleagues who wished to develop breastfeeding education and training resources specific to their geographical area. The CHO region has a population of 621,405 representing 12.4% of the Irish population, including a wide catchment of marginalised population groups (HSE, 2018). The diverse area is characterised by wide-ranging aspects of poverty, affluence and ethnicity (TUSLA/Department of Children and Youth Affairs, 2018).

### Participants

All HSE-registered General Medical Service (GMS) GPs and GPNs employed in one region in Ireland were invited to participate in the pilot study. In total, 290 GPs and 178 GPNs were identified by the HSE study gatekeeper and they received information on the study. Practitioners working in private general practice may have received communication via GP or GPN colleagues or social media to participate in the study.

### Data collection

The Strengthening the Reporting of Observational Studies (STROBE) guidelines (von Elm *et al*., 2008) were used in designing and reporting this study (Supplementary Material S1). An anonymous online survey was developed and distributed using the Qualtrics platform system. A participant information leaflet was created and distributed in advance of the study commencing. A link to the survey was distributed via an HSE HealthLink email list by the HSE study gatekeeper to all GMS GPs. GPNs registered with the HSE were informed of the study by the professional development coordinator for GPNs via email. Social media advertising was used to increase awareness of the study and encourage a good response, in addition to direct communications within GP and GPN professional groups. Data were collected from 1st November 2022 to 28th February 2023. It is acknowledged that the data collection period coincided with an increased workload in general practice due to high levels of respiratory illness in the community.

#### Survey instrument

The questionnaire used was a validated structured instrument developed by Theodoridis *et al*. (2021) enabling data collection on three domains of knowledge, attitudes and skills related to breastfeeding. The questionnaire was modified for the Irish context following a review by the CHO HSE infant feeding committee subgroup. Modifications included revised words for the Irish context, syntax and the addition of a section to explore knowledge of breastfeeding clinical skills. The final version was approved by the research team’s principal investigators (DMcG/NV) who have extensive experience in the topic area and are IBCLCs.

The questionnaire comprised four sections:

Section One sought data on demographic characteristics. No identifying information was sought and all responses were anonymous.

Section Two included 15 statements to explore knowledge on breastfeeding and utilised a five-level Likert scale.

Section Three included 16 statements to explore attitudes and perceptions towards breastfeeding and utilised a five-level Likert scale.

Section Four included four questions to explore clinical skills in relation to latch challenges, breast and nipple challenges, supplementation, supporting lactation following preterm birth and suppression of lactation following informed choice or neonatal death.

A final question sought information from participants on their subsequent education and training requirements and the preferred mode of delivery. Participants could select options from the list provided.

### Ethical approval

Low-risk ethical approval for the study was provided by the University Human Research Ethics Committee (LS-LR-22-161). Informed consent was ensured prior to engaging with the survey. Following the initial low response rate, an amendment to enable recruitment via social media was sought and approved.

### Statistical analysis

Data were analysed using SPSS. Descriptive statistics are reported and presented in tabular format. No data were inputted for the missing values and the reporting of responses is calculated on the valid denominator for each question.

## RESULTS

### Demographics

The overall response rate of the survey was 25.9% (121/468) representing 18% (53/290) of GPs and 38% (67/178) of GPNs. The survey was completed in full by 79.3% (96/121) of participants. The mean number of GPs employed per practice was 4.18 (± 2.05 SD) and GPNs 2.02 (±1.09 SD). Of the respondents, there were 14.5% (15/110) males and 85.5% (94/110) females. No responses were captured in the category ‘prefer not to say’.

The majority of the GPs and GPNs were aged between 31 and 50 years, GPs 32% (*n* = 35); GPNs 36.2% (*n* = 40). Interestingly, 23.6% (*n* = 26) of GPs graduated in the previous 15 years while 17.3% (*n* = 19) of GPNs registered/graduated in the last 15 years. In total, 26.3% (*n* = 29) of GPNs had registered/graduated over 21 years previously (Table 1).

**Table 1:** Characteristics of study participants (GPs and GPNs)

Variable		GP % (*n*)	GPN % (*n*)
Current role (*n* = 120)		44.2 (53)	55.8 (67)
Age (*n* = 110)	Under 30 years	3.6 (4)	2.7 (3)
	31–40	21 (23)	19 (21)
	41–50	11 (12)	17.2 (19)
	51–60	3.6 (4)	11 (12)
	60+	5.4 (6)	5.4 (6)
Years in current employment (*n* = 110)	Up to 5 years	20 (22)	32.7 (36)
	6–10	9.1 (10)	8.2 (9)
	11–15	3.6 (4)	4.5 (5)
	16–40	11.8 (13)	10 (11)
Years since first registration/graduation (*n* = 110)	Up to 5 years	2.7 (3)	3.6 (4)
	6–10	12.7 (14)	8.2 (9)
	11–15	8.2 (9)	5.5 (6)
	16–20	6.4 (7)	11.8 (13)
	21–30	7.3 (8)	15.4 (17)
	31+	7.3 (8)	10.9 (12)
Highest level of education (*n* = 110)	Certificate	0 (0)	7.3 (8)
	Degree	31 (34)	32.7 (36)
	Masters doctorate	9 (10)	5.5 (6)
	Other	4.5 (5)	10 (11)
^a^Professional registrations (for GPNs) (*n* = 60)	Registered general nurse	-	55.5 (61)
	Registered midwife	-	18 (20)
	Registered children’s nurse/registered prescriber	-	10 (11)

^a^Responses not mutually exclusive.

In relation to their own breastfeeding practices, over 91% (93/102) of respondents reported that they had breastfed their children or identified they intended to do so in the future (Table 2). A sizable majority 74.5%, (76/102) of participants reported always recommending breastfeeding to women, 83% (82/99) of which reported recommending a duration of breastfeeding of between 6 months and over 12 months (Table 2).

**Table 2:** Breastfeeding practice among participants

Breastfeeding practices		% (*n*)
Respondent’s children were breastfed or they intend for this in the future (*n* = 102)	Yes	91.2 (93)
	Maybe	1 (1)
	No	7.8 (8)
Duration respondent breastfed their children or intend to do so in the future (*n* = 102)	0–3 months	9.8 (10)
	3–6 months	16.7 (17)
	6–12 months	35.3 (36)
	12 + months	25.5 (26)
	Do not know	0 (0)
	N/A	12.7 (13)
Respondent recommends breastfeeding to mothers (*n* = 102)	Always	74.5 (76)
	Sometimes	21.5 (22)
	Never	4 (4)
Duration respondent recommend breastfeeding to mothers (*n* = 99)	0–3 months	0 (0)
	3–6 months	10.1 (10)
	6–12 months	46.5 (46)
	12 + months	36.4 (36)
	Do not know	7 (7)

Regarding personal confidence and ability to support mothers, respondents reported higher confidence in recognising and managing nipple problems, including mastitis and nipple thrush (52.5%, 52/99) compared to their reduced confidence in supporting mothers (Table 3) following a preterm birth (44%, 44/99) and in relation to the suppression of lactation following infant loss (44%, 44/99).

**Table 3:** Confidence in helping lactating mothers with breastfeeding-related problems

	Confident % (*n*)	Somewhat confident % (*n*)	Not confident % (*n*)
Latching problems (*n* = 99)	29.3 (29)	41.4 (41)	29.3 (29)
Recognising and managing nipple problems (e.g. mastitis and nipple thrush) (*n* = 99)	52.5 (52)	32.3 (32)	15.2 (15)
Advising on medical reasons for supplementing breastfed infants with formula milk (*n* = 99)	30.3 (30)	37.4 (37)	32.3 (32)
Supporting lactation following preterm birth (*n* = 99)	14.1 (14)	41.4 (41)	44.4 (44)
Supporting lactation suppression (e.g. following infant loss or maternal decision to stop breastfeeding) (*n* = 99)	13.1 (13)	42.4 (42)	44.4 (44)

Almost 50% of all respondents had never completed an education programme on breastfeeding (42.7%, 47/110). However, respondents either received education as part of an undergraduate programme (Table 4), 23.6% (26/110), or within a post-graduate programme, 26.3% (29/110). While a majority of respondents noted a lack of awareness of the International Code of Marketing of Breastmilk Substitutes 55.5% (61/110), 93% (92/99) were interested in completing further professional development or education on breastfeeding. Barriers identified in seeking more training included lack of time and workload (Table 4).

**Table 4:** Breastfeeding education and resources used by respondents and interest in further professional development/education on breastfeeding

		% (*n*)
^a^Any breastfeeding education programme/s completed (*n* = 110)	As part of your undergraduate degree (medicine or nursing)/nurse education training programme	23.6 (26)
	As part of a post-graduate education programme	26.3 (29)
	Breastfeeding practical hands-on skills training programme	21.8 (24)
	Continuous professional development programme (not assessed)	16.3 (18)
	WHO 20-hour breastfeeding programme	6.3 (7)
	HSELand infant feeding eLearning modules	12.7 (14)
	Never completed any breastfeeding education programme	42.7 (47)
^a^Resources/tools respondents use in practice to support breastfeeding (*n* = 110)	Signposting families to HSE mychild.ie	48 (53)
	Signposting to HSE ‘My Child: 0-2 years- Expert advice for every step’ book	26.3 (29)
	Mychild.ie for updating my professional knowledge	14.5 (16)
	Breastfeeding Observation and Assessment Tool (BOAT)	4.5 (5)
	National Infant Feeding Policy for Primary Care Teams and CHOs (2019)	6.3 (7)
	The WHO BFHI ten steps to successful breastfeeding infographic	6.3 (7)
	Referral to HSE PHN IBCLC (lactation consultant)	36.3 (40)
	Referral to HSE PHN-led support group	38 (42)
	Referral to Maternity Hospital IBCLC	23.6 (26)
	Referral to private IBCLC	26.3 (29)
	Signposting or referral to voluntary breastfeeding support	40 (44)
	Breast milk sample facilities	2.7 (3)
Respondent aware of the International Code of Marketing of Breastmilk Substitutes (*n* = 110)	Yes	44.5 (49)
	No	55.5 (61)
For those aware, if the respondent adheres to the International Code of Marketing of Breastmilk Substitutes (*n* = 49)	Yes	75.5 (37)
	No	2 (1)
	Unsure	22.5 (11)
^a^Respondent interested in completing further professional development and education on breastfeeding (*n* = 99)	Yes, education on current recommendations (evidence-based)	80.8 (80)
	Yes, practical skills training on how to support mothers to feed effectively	62.6 (62)
	Yes, accredited to a university or professional body	0 (0)
	No	7 (7)
^a^Method/s of training respondent most interested in (*n* = 50)	Online	32 (16)
	Face to face	16 (8)
	Blended	80 (40)
^a^Barrier/s preventing respondent from getting involved in training (*n* = 99)	Time	84.7 (83)
	Workload	71.7 (71)
	Cost	29.3 (29)
	Other	6 (6)

^a^Responses not mutually exclusive.

A variety of information sources provided by the HSE were accessed by respondents to support parents and breastfeeding, as noted in Table 4, including the website mychild.ie, and the National Infant Feeding Policy for Primary Care Teams and CHOs.

Table 5 presents the confidence, attitudes and perceptions of both groups to support breastfeeding mothers. While 56% (57/102) described themselves as confident with their knowledge about breastfeeding, 76% (78/102) said they attained the majority of their knowledge through personal research and a significant majority identified that their knowledge could be improved 90.2% (92/102).

**Table 5:** Knowledge/confidence of breastfeeding support and attitude/perception towards breastfeeding

	Strongly disagree % (*n*)	Disagree % (*n*)	Neither agree nor disagree % (*n*)	Agree % (*n*)	Strongly agree % (*n*)
I am confident with my knowledge about breastfeeding (*n* = 102)	8.8 (9)	18.6 (19)	16.7 (17)	44.1 (45)	11.7 (12)
I have obtained most of my knowledge about breastfeeding through my own personal research (*n* = 102)	1.9 (2)	9.8 (10)	11.8 (12)	55 (56)	21.5 (22)
My level of knowledge could be improved (*n* = 102)	1.9 (2)	1.9 (2)	5.9 (6)	54 (55)	36.2 (37)
I am confident that I can manage breastfeeding-related issues in my everyday practice (*n* = 102)	6.9 (7)	15.7 (16)	21.5 (22)	47 (48)	8.8 (9)
Formula milk is easier to digest than maternal milk (*n* = 102)	49 (50)	38.2 (39)	10.8 (11)	0.9 (1)	0.9 (1)
A breastfeeding mother should avoid alcohol (*n* = 102)	4.9 (5)	22.5 (23)	16.7 (17)	38.2 (39)	17.6 (18)
A carrier of hepatitis B who has been vaccinated can safely breastfeed (*n* = 102)	2.9 (3)	1.9 (2)	23.5 (24)	55.9 (57)	15.7 (16)
A carrier of HIV can transfer the virus to her baby through breastfeeding (*n* = 102)	5.9 (6)	32.3 (33)	32.3 (33)	21.5 (22)	7.8 (8)
A mother with a fever > 38°C should temporarily interrupt breastfeeding (*n* = 102)	24.5 (25)	43 (44)	15.7 (16)	15.7 (16)	0.9 (1)
A mother with mastitis should stop breastfeeding (*n* = 102)	48 (49)	40.2 (41)	4.9 (5)	5.9 (6)	0.9 (1)
Breastfeeding should continue if the mother smokes (*n* = 102)	3.9 (4)	10.8 (11)	21.5 (22)	47 (48)	16.7 (17)
I am confident discussing safe medication use with breastfeeding mothers (*n* = 102)	3.9 (4)	21.5 (22)	22.5 (23)	45 (46)	6.9 (7)
Breast surgeries i.e. augmentation or reduction make breastfeeding difficult (*n* = 102)	2.9 (3)	18.6 (19)	47 (48)	29.4 (30)	1.9 (2)
Breastfed babies are less likely to suffer reflux (*n* = 102)	5.9 (6)	10.8 (11)	30. 4 (31)	45 (46)	7.8 (8)
Breastfeeding mothers need to night wean at 6 months (*n* = 102)	45 (46)	36.3 (37)	14.7 (15)	1.9 (2)	1.9 (2)
Attitude and perception towards breastfeeding					
Respondent in favour of exclusive breastfeeding (*n* = 99)	1 (1)	8 (8)	21.2 (21)	50.5 (50)	19.2 (19)
Respondent in favour of breastfeeding combined with formula milk (*n* = 99)	3 (3)	14.1 (14)	40.4 (40)	38.4 (38)	4 (4)
Respondent in favour of breastfeeding in public (*n* = 99)	0	1 (1)	1 (1)	37.4 (37)	60.6 (60)
Respondent in favour of breastfeeding while returning to work (*n* = 99)	0	2 (2)	3 (3)	50.5 (51)	43.4 (43)
Breastfeeding has an impact on the social life of a mother (*n* = 99)	5 (5)	17.2 (17)	19.2 (19)	44.4 (44)	14.1 (14)
Breastfeeding has an impact on the professional life of a mother (*n* = 99)	4 (4)	18.2 (18)	10 (10)	51.5 (51)	16.2 (16)
Breastfeeding makes the father/partner feel isolated from raising their child (*n* = 99)	24.2 (24)	35.4 (35)	28.3 (28)	12.1 (12)	0
A daily formula milk top-up has an impact on exclusive breastfeeding	3 (3)	18.2 (18)	27.3 (27)	41.4 (41)	10.1 (10)
Breastfeeding is more convenient and cheaper than formula milk	2 (2)	0	11.1 (11)	38.4 (38)	48.5 (48)
Mothers with excess milk should be encouraged to donate their milk to maternal/donor milk banks	0	4 (4)	45.5 (45)	39.4 (39)	11.1 (11)
Respondent has time to inform antenatal pregnant women about the importance of breastfeeding/risks of not breastfeeding	7 (7)	27.3 (27)	23.2 (23)	37.4 (37)	5 (5)
Respondent feels low breastfeeding rates in Ireland are due to healthcare professionals not informing mothers about breastfeeding	7 (7)	37.4 (37)	35.4 (35)	17.2 (17)	3 (3)

Almost 70% (69/99) of respondents strongly agreed or agreed with exclusive breastfeeding (feeding an infant only breastmilk) while 42.4% (42/99) favoured breastfeeding combined with commercial milk formula. Participants were asked if a daily formula milk top-up had an impact on breastfeeding and 51.5% (51/99) agreed or strongly agreed with this statement, while 27.3% (27/99) neither agreed nor disagreed. A majority agreed that breastfeeding should be continued once the mother returns to work, and 67.7% (67/99) of respondents agreed that breastfeeding has an impact on the professional life of a mother (Table 5).

## DISCUSSION

The aim of this pilot study was to establish the breastfeeding KAPs of GPs and GPNs in one CHO in Ireland. It is recognised that breastfeeding and lactation challenges can develop in the post-natal period and support from healthcare professionals is imperative to achieve positive breastfeeding outcomes (Brown, 2017; Feldman Winter *et al*., 2020; Buckland *et al*., 2022; Scime *et al*., 2023).

Previous Irish research by Finneran and Murphy (2004) identified that less than 10% of GPs had received formal breastfeeding education lectures at the undergraduate level. Ten years later, Whelan and Kearney (2015) explored healthcare professionals’ and women’s views of breastfeeding support in Ireland. Unsurprisingly, midwives and public health nurses expressed more confidence in supporting breastfeeding mothers than medical doctors. Similar to our pilot study in one CH0, confidence was higher among those who had personal experience of breastfeeding or had received breastfeeding education. Whelan and Kearney’s (2015) study notes women’s challenge when they had to seek breastfeeding support elsewhere, and if inaccurate information was received from an HCP, particularly their GP. More recently, McCarthy *et al*. (2021) explored post-graduate medical education in Ireland specifically in relation to medication use in pregnancy and lactation and identified 18 post-graduate medical programmes that do not mention lactation and breastfeeding.

Theodoridis *et al*. (2021) identified that participants lacked knowledge in relation to the safe use of medication for mothers during breastfeeding. Breastfeeding mothers may present to their GP and GPN with breastfeeding conditions such as mastitis, Raynaud’s phenomenon or candida, where medication is indicated and knowledge of the pharmacological transfer of drugs in breastmilk is important to inform evidence-based care.

Consistent evidence reported over 15 years suggests education on infant feeding is limited in primary and secondary care (Smale *et al*., 2006; Pound *et al*., 2014; Webber and Serowoky, 2017; Holtzman and Underwood, 2018). Walsh *et al*. (2023) reported that Irish public health nurses (PHNs) were confident with some aspects of breastfeeding management; however, they experienced low confidence in supporting mothers with lactation following preterm birth, breast surgery and in the use of a breast pump. Thompson *et al*. (2020) describe how women based in the UK perceived HCPs’ disapproval of breastfeeding beyond infancy, which in turn fostered a hesitancy to seek advice from HCPs. Canadian physicians also reported a lack of knowledge and confidence in supporting breastfeeding during the newborn period in relation to latch assessment and delayed lactogenesis (Baerg *et al*., 2021).

It is also pertinent to mention the topic of exclusive breastfeeding, given the prevalence of a formula milk culture in Ireland. The majority of participants in our study supported exclusive breastfeeding as recommended by the WHO (2023) and were aware that breastfeeding was more convenient and cheaper than CMF. There was a degree of uncertainty about CMF use, consistent with the findings of Brodribb *et al*. (2008). We also report uncertainty among GPs’ and GPNs’ knowledge in relation to the International Code of Marketing of Breastmilk Substitutes.

The results of this pilot study, albeit with a low response rate, indicate that the majority of GPs and GPNs have not completed a breastfeeding education programme, noting that they have improved their knowledge base via personal learning. These results are consistent with previous studies noting the variable and ad hoc preparation of students to support breastfeeding and lactation (Biggs *et al*., 2020; Becker *et al*., 2021; Kinoshita *et al*. 2021; Theodoridis *et al*., 2021).

The WHO (2023) recommends that breastfeeding is the ideal method of infant feeding and should continue for the first 2 years of life and this research highlights the challenges for GPs and GPNs in promoting breastfeeding antenatally and supporting families on their breastfeeding journey. In light of the current deficit in the preparedness of GPs and GPNs, it is important that at the university level, medical and nursing schools revise their curriculum to ensure adequate breastfeeding and lactation education inclusive of clinical skills exposure for students (Biggs *et al*., 2020; Blitman *et al*., 2022). This investment in public health education will support and protect breastfeeding for women, infants and families at the societal level (Brown, 2017); notwithstanding improved nutrition, healthy weight status and approaches to managing chronic disease (Bartik *et al*., 2013; Lee *et al.*, 2022; Stordal, 2023).

## LIMITATIONS

The survey was distributed during the winter months when there was a higher incidence of respiratory infection in the community with resultant busier clinics for the GPs and GPNs. This may have contributed to the lower response rate. Competing research demands for participation in online surveys may have impacted our response rate, given the additional workload for GPs and GPNs. We are grateful to those who participated in this study.

The pilot survey was limited to one CHO area which may have unique characteristics and contexts and the results may not be generalisable outside of the region; however, the findings are consistent with earlier Irish data and published international literature. There may be a risk of bias with those participants who had breastfed or were interested in the topic of breastfeeding and lactation and decided to complete the survey. Despite the limitations the results of the study are important and they have prompted the HSE and Irish College of General Practitioners to complete a national survey of GPs, GPNs and GP trainees. A qualitative study is also indicated in view of the small response rate to enable a wider discussion of key points.

Despite the limitations noted, this study reports PPI engagement and co-designed methods thus strengthening the research. The inclusion of PPI stakeholders in developing public health measures enables shared decision-making and tailoring interventions for specific communities (Fouladi *et al*., 2023).

## CONCLUSION

This cross-sectional co-designed PPI study identifies inconsistent knowledge and attitudes among GPs and GPNs to breastfeeding and indicates opportunities to engage in co-developing learning resources. The study identified a positive breastfeeding culture with GPs and GPNs in this CHO region recommending breastfeeding. The content and delivery of breastfeeding programmes should be reviewed with urgent development of theoretical and skills-based training for medical and nursing programmes at undergraduate and post-graduate levels. The results indicate a gap in knowledge and highlight a need for further research and education to ensure that every child is given the best start in life.

## Supplementary Material

daae021_suppl_Supplementary_Material
